# Social inequalities in the use of online food delivery services and associations with weight status: cross-sectional analysis of survey and consumer data

**DOI:** 10.1136/bmjph-2023-000487

**Published:** 2024-07-24

**Authors:** Steven Cummins, Alexandra Irene Kalbus, Laura Cornelsen, Jean Adams, Emma Boyland, Thomas Burgoine, Cherry Law, Frank de Vocht, Martin White, Amy Yau

**Affiliations:** 1 Population Health Innovation Lab, London School of Hygiene & Tropical Medicine, London, UK; 2 MRC Epidemiology Unit, University of Cambridge, Cambridge, UK; 3 Department of Psychology, University of Liverpool, Liverpool, UK; 4 Department of Agri-Food Economics and Marketing, University of Reading, Reading, UK; 5 Population Health Sciences, Bristol Medical School, University of Bristol, Bristol, UK

**Keywords:** Food Services, Supermarkets, Sociodemographic Factors, Body Mass Index

## Abstract

**ABSTRACT:**

**Background:**

Little is known about who uses online food delivery services and how use of these services is associated with social inequalities in food purchasing and diet-related health. This study explored associations between social position and use of online takeaway food and grocery delivery services, and its association with weight status.

**Methods:**

Data were obtained from households in a consumer research panel living in London and the north of England (n=1521) in February 2019. Use of online grocery delivery services was determined via recorded purchases, and takeaway food delivery app use via survey responses. Social position was approximated through occupation-based social grade and household income. We used logistic regression to estimate the association between social position and use of online delivery services, and the relationship between online delivery service use and weight status.

**Results:**

Overall, 13.2% of respondents used takeaway food delivery apps over a 7-day period and 15.6% of households used online grocery delivery services over a 4-week period. High-income households were more likely to use online grocery delivery services than low-income households (OR 2.01, 95% CI 1.22 to 3.34). In contrast, households with lower social grade were more likely to use takeaway food delivery apps compared with households in the highest grade (OR 2.31, 95% CI 1.38 to 3.87). While takeaway food delivery app use was positively associated with living with obesity (relative risk ratio 1.84, 95% CI 1.20 to 2.82), use of online grocery delivery services was not.

**Discussion:**

Findings indicate that use of online food delivery services is patterned by markers of social position and weight status, which may lead to dietary inequalities. The potential impact of increased and differential usage of online delivery services on diet and dietary inequalities warrants further research.

**WHAT IS ALREADY KNOWN ON THIS TOPIC**

- Previous research suggests that while purchasing groceries online is associated with healthier food purchasing, use of online takeaway food delivery apps tends to promote less healthy food purchasing. This study investigated whether use of online food delivery services was patterned by markers of social position (income and occupational social grade), and whether use of these services was associated with weight status.

**WHAT THIS STUDY ADDS**

- Use of online grocery delivery services was associated with higher household income, but not with social grade and weight status. Use of online takeaway food delivery apps was associated with lower occupational social grade and higher likelihood of living with obesity, but not with income.

**HOW THIS STUDY MIGHT AFFECT RESEARCH, PRACTICE OR POLICY**

- The differential use of online food delivery services may exacerbate dietary inequalities and warrants further research.

## Introduction

 Purchases from food retailers are the main way in which consumers obtain food, making them one of the key drivers of population diet.^1^ Diets with high intakes of sugars, salt and saturated fats, as well as low intakes of fruit, vegetables and fibre, are key risk factors for obesity, diabetes and associated non-communicable diseases globally.^2^ In the UK, 28% of the adult population and 16% of those aged 2–15 years were living with obesity in 2019^3^ and dietary risks account for 15% of non-communicable disease mortality.^2^ Diet and dietary health are further unequally distributed across the population, with socioeconomically disadvantaged groups at higher risk of suffering diet-related illness.^4^

The in-person purchasing of groceries from supermarkets and convenience stores and the in-person purchasing of takeaway foods (prepared meals and snacks from fast-food outlets, takeaways and restaurants) has traditionally been the main way by which households acquire food. However, food retailing in the grocery and out-of-home food sectors has been undergoing a transformation. Digital on-demand technology has rapidly reshaped food distribution and delivery, making grocery and prepared takeaway meals more accessible and convenient.^5 6^ Online grocery delivery in the UK is not new and was pioneered by some major supermarket chains over 20 years ago. However, the recent rapid increase in the ubiquity of home and mobile internet access, development, ownership and use of smartphones and apps, and growing consumer adoption of e-commerce have promoted the use of online food delivery services. This allowed technology-led ‘disruptor’ food companies such as Ocado, Deliveroo, Uber Eats and Just Eat to gain entry into both the grocery and takeaway food retail market in the UK. These ‘digital-first’ companies primarily operate as either online platforms that directly sell and deliver food (such as Ocado) or as marketplace aggregators and logistics partners that give both chain and independent food businesses access to a third-party delivery network (such as Just Eat). These companies do not only directly change how consumers access food but also accelerate the entry of existing physical food retailers into the digital market.^7^ As a result, this has increased the number of food retailers who are able to offer delivery services and have expanded the number and range of grocery and takeaway food options available to consumers.^8^

How these changes affect inequalities in food purchasing, diet and diet-related disease is unknown. In the grocery sector, online purchases may result in a healthier overall basket as users of digital services may be less influenced by in-store marketing and promotions.^9 10^ However, high minimum spend requirements as well as delivery costs coupled with reductions in the cost of bulk buying means there is potential for excess purchases.^11^ This may lead to over-consumption, food waste or an increase in purchases of shelf-stable and processed products.^12^ Online grocery purchasing has previously been associated with having higher education and income.^13^

Within the takeaway food sector, defined as fast-food outlets, takeaways and restaurants offering prepared meals and food for consumption off the premises, the increasing availability of food delivery services has expanded the number of restaurants able to offer delivery, increased the reach of individual restaurants and meal options available to consumers, and reduced the effort and time required to purchase takeaway food.^14^ Previous research noted an unclear relationship between markers of social position and use of online food delivery apps in the UK,^15^ while international research reported greater odds of using these services associated with higher levels of income and education.^16^ Recent research on fast-food delivery services found that the meal options available were primarily unhealthy,^8 17^ and that the majority of marketing strategies on these platforms concerned unhealthy food and drink items.^18^ Increased access to these meals as well as other takeaway foods, which already tend to be higher in fat, salt, sugar and energy,^19 20^ may therefore negatively affect diet quality. Increased purchases of these delivered foods may also replace home-prepared foods, which are often healthier.^21^ The COVID-19 pandemic has resulted in an acceleration in the use of both grocery and takeaway food delivery services,^22 23^ meaning that further research in this area is needed.

To improve our understanding of the impact of ongoing changes to the food retail system, an important first step is to investigate who uses online food delivery services and whether use of these services is associated with diet-related disease. In this article we use data from a large consumer panel and a survey conducted among said panel to begin to answer these questions. We use the term ‘online grocery delivery’ to describe online purchases of groceries from supermarkets and convenience stores for ‘click-and-collect’ or home delivery. We define ‘online takeaway food delivery’ as the online purchase of ready-to-eat food direct from a takeaway or restaurant or via a third-party aggregator or delivery partner such as Just Eat or Deliveroo. As with groceries this can include purchases for both ‘click-and-collect’ and delivery. First, we explore whether there are associations between social position and use of online food delivery services for both groceries and takeaway food. Second, we investigate whether use of these services is associated with weight status proxied through self-reported body mass index (BMI).

## Methods

### Study design and sample

In this cross-sectional study, we accessed data from the Transport for London Study which evaluated the impact of the removal of high fat, salt and sugar food advertising on the Transport for London network.^24^ Data are from a sample of households living in London and the north of England drawn from the GB Kantar Fast Moving Consumer Goods (FMCG) panel (n=1557 households). Households in this FMCG panel are representative of the regions from which they are drawn on the basis of household size, number of children, occupational socioeconomic status and age of the main food shopper, with the latter denoting the household’s primary food shopper and reporter. Panel households are recruited by Kantar through post and email, and sample representativeness is assessed every 4 weeks.^25^ We had two types of data available for this sample. First, objective item-level daily food and beverage purchases by these panellists between June 2018 and July 2019 (used to determine the use of online grocery purchasing); and second, self-reported data from a bespoke survey conducted among the panellists in February 2019 (used to determine online takeaway delivery service use).^26 27^

### Grocery purchasing data

Households are recruited to the Kantar FMCG panel to provide data on their day-to-day food and beverage purchases for consumption at home. The main food shopper in each household records purchases using a hand-held barcode scanner. Non-barcoded products such as loose fruits and vegetables are recorded using bespoke barcodes. Participants additionally provide information from receipts. Purchases cover a range of grocery retailers such as supermarkets (including online), convenience stores, corner shops, specialist stores and markets.

Grocery purchases were coded as online or in-store according to a proprietary classification. Online grocery purchases covered deliveries and click-and-collect occasions from the following retailers: Tesco, Asda, Morrison, Ocado, Sainsbury, Waitrose, Marks and Spencer, Iceland, Wilko, Superdrug, Boots and miscellaneous internet sources. To keep the analysis of online grocery and takeaway delivery service use consistent, we used grocery purchase data for 1 month (February 2019) which matches the time period of the survey data. We then created the binary variable ‘online grocery delivery service use’ which was coded as 1 if households had made at least one online grocery purchase, defined as delivery and/or click-and-collect, in February 2019, and zero if otherwise.

### Survey data on online takeaway food delivery service use

The main shoppers in each household were asked to complete a short bespoke online survey, including a question on their use of mobile applications (apps) for takeaway food delivery. The survey took place over a 10-day period in February 2019 and was administered by Kantar. To understand takeaway purchases, respondents were asked: ‘In the past 7 days, how many times, if at all, did you use the following food delivery apps?’ Responses were given for the categories: Just Eat, Deliveroo, Uber Eats, and Other. The category ‘Other’ included company-specific services (chain, for example, Domino’s, and non-chain) rather than aggregators. This variable was then used to derive a binary response variable: usage of takeaway food delivery apps at least once in the past 7 days (yes/no). We used dichotomised outcomes for both online grocery and takeaway food delivery use due to their low frequency (see Results) and positively skewed distributions among users.

### Sociodemographic characteristics

Household sociodemographic characteristics are self-reported and collected by Kantar annually. Participants’ social position was characterised as both household income and household main food shopper’s occupational social grade, referred to as social grade. Social grade was categorised using the National Readership Survey classification (A, B, C1, C2, D, E).^28^ Accordingly, we determined four groups: High (AB: higher and intermediate managerial, administrative, and professional), middle-high (C1: supervisory, clerical and junior managerial, administrative and professional), middle-low (C2: skilled manual workers), and low (DE: semi-skilled and unskilled manual workers, state pensioners, casual and lowest grade workers, unemployed with state benefits only). Self-reported household income was measured in three bands: £0–19,999, £20 000–49 999, and £50 000 or more per annum. We chose these two indicators of social position, and analysed them separately, as income has been previously associated with online grocery delivery service use^13^ but is not known for all study households. Occupational social grade was known for all studied households and has been found to be associated with purchasing behaviour.^29^ Covariates hypothesised to confound any associations were: number of adults and children (<16 years of age) in the household, region (London, north of England), age (in 10-year age bands), sex (male/female), and working status of the main household food shopper. We categorised working status into six categories: full-time employee, part-time employee, self-employed, retired, not looking for work or unable to work (looking after home or family, long-term sick or disabled, away from work due to illness, maternity leave, holiday or unemployed and not looking for work), and other (government-sponsored training scheme, other paid work, student, actively looking for paid work or other).

### Weight status

Kantar collects self-reported height and weight for the main household food shopper on an annual basis. Data were available for 1245 households (81.9%). BMI was then calculated using the standard equation (weight (kg)/height (m^2^)) and classified into three weight status categories, with underweight and healthy weight combined due to the low prevalence of underweight (n=30, 2%)^30^: underweight and healthy weight: <25 kg/m^2^; overweight: 25–29.9 kg/m^2^; and obesity: ≥30 kg/m^2^.

### Statistical analysis

We provide summary statistics of sample characteristics. Using binary logistic regression models, we estimated odds ratios (OR) with 95% confidence intervals (95% CI) for the association between social position and the use of online grocery or takeaway food delivery services. First, we ran separate unadjusted models to explore associations of social grade and income with both online food delivery variables. Second, we adjusted these models for relevant sociodemographic variables. Third, we used multinomial logistic regression to estimate the relative risk ratio (RRR) of having overweight or obesity in relation to online grocery delivery service and takeaway food delivery app use while adjusting for sociodemographic characteristics.

For the analyses of use of online grocery delivery services, we excluded households that had not reported any grocery food shopping during the 4-week study period (n=36). To facilitate comparability, we restricted the analysis of online takeaway food delivery service use to the same households, resulting in an analytical sample of n=1521 households. Because income and weight status were not known for all respondents (15% and 18% missing observations, respectively), we tested whether ‘missingness’ was associated with online food delivery service use which would inhibit dropping missing observations. We did this by creating a binary variable of missing observations for both income and weight status and we then regressed this against both online grocery and takeaway delivery service use and other covariates (see online supplemental material tables S1, S2). As no statistically significant associations for both online grocery delivery service or takeaway food delivery app use were found, we proceeded with complete case analyses in models including income and/or weight status. All analyses were conducted in Stata IC v.16.

## Results

A summary of sample characteristics is provided in table 1. In February 2019, 15.6% of households purchased groceries online at least once, 13.2% reported having used takeaway food delivery apps in the 7 days before the survey, and 3.5% used both online food delivery services.

**Table 1:** Sample characteristics, stratified by online food delivery service use

	Total% (n)	Used online grocery delivery service*% (n)	Used online takeaway food delivery app†% (n)
Analytical ample	100 (1521)	15.6 (237)	13.2 (201)
Sex			
Male	28.3 (431)	19.8 (47)	28.9 (58)
Female	71.7 (1090)	80.2 (190)	71.1 (143)
Age (years)			
20–29	4.3 (66)	4.2 (10)	10.5 (21)
30–39	15.7 (238)	16.9 (40)	25.9 (52)
40–49	22.8 (347)	33.3 (79)	33.8 (68)
50–59	25.7 (391)	23.6 (56)	18.9 (38)
60–69	19.3 (293)	13.9 (33)	6.5 (13)
70+	12.2 (186)	8.0 (19)	4.5 (9)
Social grade‡			
High (AB)	22.0 (335)	24.5 (58)	17.9 (36)
Middle-high (C1)	43.7 (664)	40.5 (96)	39.3 (79)
Middle-low (C2)	16.1 (245)	17.3 (41)	18.9 (38)
Low (DE)	18.2 (277)	17.7 (42)	23.9 (48)
Income (per annum)			
Up to £19 999	21.5 (327)	16.9 (40)	10.5 (21)
£20 000–49 999	43.7 (665)	40.5 (96)	18.9 (38)
£50 000 or more	19.5 (296)	27.4 (65)	49.8 (100)
Unknown/missing	15.3 (233)	15.2 (36)	20.9 (42)
Employment			
Full time	39.1 (594)	38.8 (92)	51.2 (103)
Part time	14.4 (219)	13.9 (33)	14.4 (29)
Self-employed	8.5 (129)	8.4 (20)	9.0 (18)
Retired	22.4 (340)	16.5 (39)	8.0 (16)
Looking after home/family	7.2 (109)	6.8 (16)	6.5 (13)
Other§	8.6 (130)	15.6 (37)	11.0 (22)
Region			
North	54.4 (828)	48.5 (115)	52.2 (105)
London	45.6 (693)	51.5 (122)	47.8 (96)
Weight status¶			
Underweight and healthy weight	32.8 (499)	32.5 (77)	29.9 (60)
Overweight	27.6 (420)	20.7 (49)	27.9 (54)
Obesity	21.4 (326)	25.3 (60)	27.9 (54)
Missing	18.2 (276)	21.5 (51)	16.4 (33)
Number of adults, average (SD)	2.05 (0.87)	2.11 (0.84)	2.11 (0.87)
Number of children, average (SD)	0.46 (0.84)	0.62 (0.91)	0.72 (1.01)

* During the month of February 2019; .

† During the previous 7 days days (in February 2019); .

‡ Social grade was based on the National Readership Survey classification (National Readership Survey, 2018): High (AB: higher and intermediate managerial, administrative and professional), middle-high (C1: supervisory, clerical and junior managerial, administrative and professional), middle-low (C2: skilled manual workers), and low (DE: semi-skilled and unskilled manual workers, state pensioners, casual and lowest grade workers, unemployed with state benefits only); .

§ On a government -sponsored training scheme; working paid or unpaid for your own or family’s business; away from work ill, on maternity leave, on holiday or temporarily; laid off; doing any other kind of paid work; retired; student; long term sick or disabled; actively looking for paid work; unemployed and not looking for work; none of the above; .

¶ Living with uUnderweight and healthy weight is defined as BMI <25 kg/m2, overweight as BMI kg/m25–29.9 kg/m2, and obesity as BMI ≥30 kg/m2.

### Online grocery delivery service use

In fully adjusted models (table 2, column 3), there was no association between social grade and using online grocery delivery services. When considering household income instead of social grade (table 2, column 4), those with highest incomes had twice the odds of purchasing groceries online compared with those in the lowest income group (OR 2.01, 95% CI 1.22 to 3.34).

**Table 2:** Associations between social class and use of online grocery delivery services in the previous month

	Unadjusted (social grade)		Unadjusted (income)		Adjusted (social grade)		Adjusted (income)	
	OR (95% CI)	P	OR (95% CI)	P	OR (95% CI)	P	OR (95% CI)	P
Social grade*								
High (AB)	Ref		X		Ref		X	
Middle-high (C1)	0.81 (0.57 to 1.15)	0.24	X		0.80 (0.56 to 1.16)	0.24	X	
Middle-low (C2)	0.96 (0.62 to 1.49)	0.86	X		0.98 (0.62 to 1.54)	0.93	X	
Low (DE)	0.85 (0.55 to 1.32)	0.47	x		0.74 (0.46 to 1.19)	0.21	X	
Income (per annum)								
Up to £19 999	X		Ref		X		Ref	
£20 000–49 999	X		1.21 (0.82 to 1.80)	0.34	X		1.33 (0.86 to 2.05)	0.19
£50 000 or more	X		2.02 (1.31 to 3.10)	<0.01	X		2.01 (1.22 to 3.34)	<0.01
Sex								
Female					Ref		Ref	
Male					0.58 (0.41 to 0.83)	<0.01	0.57 (0.39 to 0.84)	<0.01
Age (years)								
20–29					Ref		Ref	
30–39					1.14 (0.52 to 2.49)	0.74	1.07 (0.48 to 2.36)	0.87
40–49					1.73 (0.83 to 3.63)	0.14	1.64 (0.77 to 3.48)	0.20
50–59					1.05 (0.49 to 2.24)	0.90	1.09 (0.50 to 2.34)	0.83
60–69					0.74 (0.31 to 1.77)	0.50	0.81 (0.33 to 2.01)	0.65
70+					0.64 (0.23 to 1.81)	0.40	0.97 (0.31 to 3.04)	0.96
Number of adults					1.04 (0.88 to 1.23)	0.64	1.01 (0.84 to 1.23)	0.89
Number of children					1.12 (0.93 to 1.35)	0.22	1.11 (0.90 to 1.35)	0.33
Employment								
Full time					Ref		Ref	
Part time					0.87 (0.54 to 1.37)	0.54	0.86 (0.52 to 1.41)	0.55
Self-employed					0.94 (0.55 to 1.62)	0.82	0.89 (0.78 to 1.64)	0.70
Retired					1.36 (0.70 to 2.64)	0.36	0.98 (0.44 to 2.17)	0.95
Looking after home/family					0.78 (0.42 to 1.45)	0.43	1.13 (0.59 to 2.16)	0.70
Other†					2.40 (1.48 to 3.87)	<0.01	2.52 (1.51 to 4.22)	<0.01
Region								
North					Ref		Ref	
London					1.34 (1.00 to 1.78)	0.05	1.21 (0.88 to 1.66)	0.23
Number of observations	1521		1288		1521		1288	
Log likelihood	−657.26		−551.82		−630.77		−531.07	

* Social grade was based on the National Readership Survey classification (National Readership Survey, 2018): High (AB: higher and intermediate managerial, administrative and professional), middle-high (C1: supervisory, clerical and junior managerial, administrative and professional), middle-low (C2: skilled manual workers), and low (DE: semi-skilled and unskilled manual workers, state pensioners, casual and lowest grade workers, unemployed with state benefits only); .

† On a government -sponsored training scheme; working paid or unpaid for your own or family’s business; away from work ill, on maternity leave, on holiday or temporarily; laid off; doing any other kind of paid work; retired; student; long term sick or disabled; actively looking for paid work; unemployed and not looking for work; none of the above.

### Takeaway food delivery app use

After adjusting for sociodemographic characteristics, lower social grade was associated with the use of takeaway food delivery apps (table 3, column 3). In comparison to the highest social grade, respondents with the lowest social grade had more than twice the odds of using these services (OR 2.31, 95% CI 1.38 to 3.87). Furthermore, respondents with middle-low social grade had 69% greater odds of using takeaway food delivery apps (OR 1.69, 95% CI 1.01 to 2.84). In contrast to online grocery shopping, takeaway food delivery app use was not associated with income (table 3, column 4).

**Table 3:** Associations between social class and use of online takeaway delivery services in the previous 7 days

	Unadjusted (social grade)		Unadjusted (income)		Adjusted (social grade)		Adjusted (income)	
	OR (95% CI)	P	OR (95% CI)	P	OR (95% CI)	P	OR (95% CI)	P
Social grade*								
High (AB)	Ref		X		Ref		X	
Middle-high (C1)	1.12 (0.74 to 1.70)	0.59	X		1.19 (0.77 to 1.84)	0.43	X	
Middle-low (C2)	1.52 (0.93 to 2.49)	0.09	X		1.69 (1.01 to 2.84)	0.05	X	
Low (DE)	1.74 (1.09 to 2.77)	0.02	x		2.31 (1.38 to 3.87)	<0.01	X	
Income (per annum)								
Up to £19 999	X		Ref		X		Ref	
£20 000–49 999	X		1.35 (0.90 to 2.01)	0.15	X		1.03 (0.66 to 1.62)	0.88
£50 000 or more	X		1.26 (0.79 to 2.01)	0.34	X		0.68 (0.39 to 1.18)	0.17
Sex								
Female					Ref		Ref	
Male					1.16 (0.81 to 1.66)	0.42	1.12 (0.77 to 1.64)	0.56
Age (years)								
20–29					Ref		Ref	
30–39					0.66 (0.35 to 1.24)	0.30	0.59 (0.31 to 1.13)	0.11
40–49					0.55 (0.30 to 1.00)	0.05	0.58 (0.30 to 1.03)	0.06
50–59					0.25 (0.13 to 0.48)	<0.001	0.27 (0.14 to 0.52)	<0.001
60–69					0.12 (0.05 to 0.29)	<0.001	0.11 (0.04 to 0.30)	<0.001
70+					0.13 (0.04 to 0.47)	<0.01	0.14 (0.04 to 0.55)	<0.01
Number of adults					1.06 (0.88 to 1.27)	0.54	1.07 (0.88 to 1.31)	0.48
Number of children					1.10 (0.92 to 1.32)	0.31	1.10 (0.90 to 1.34)	0.35
Employment								
Full time					Ref		Ref	
Part time					0.65 (0.40 to 1.06)	0.08	0.68 (0.41 to 1.14)	0.15
Self-employed					0.76 (0.43 to 1.35)	0.35	0.70 (0.37 to 1.32)	0.27
Retired					0.73 (0.29 to 1.87)	0.52	0.73 (0.25 to 2.20)	0.58
Looking after home/family					0.47 (0.24 to 0.92)	0.03	0.54 (0.26 to 1.13)	0.10
Other†					0.74 (0.42 to 1.31)	0.31	0.88 (0.49 to 1.59)	0.67
Region								
North					Ref		Ref	
London					1.16 (0.85 to 1.59)	0.34	1.08 (0.78 to 1.52)	0.62
Number of observations	1521		1288		1521		1288	
Log likelihood	−589.99		−519.91		−542.63		−482.87	

* Social grade was based on the National Readership Survey classification (National Readership Survey, 2018): High (AB: higher and intermediate managerial, administrative and professional), middle-high (C1: supervisory, clerical and junior managerial, administrative and professional), middle-low (C2: skilled manual workers), and low (DE: semi-skilled and unskilled manual workers, state pensioners, casual and lowest grade workers, unemployed with state benefits only); .

† On a government -sponsored training scheme; working paid or unpaid for your own or family’s business; away from work ill, on maternity leave, on holiday or temporarily; laid off; doing any other kind of paid work; retired; student; long term sick or disabled; actively looking for paid work; unemployed and not looking for work; none of the above.

### Associations between online food delivery service use and weight status

Adjusted multinomial regression models did not reveal associations between the use of online grocery delivery and weight status (table 4). Compared with those who did not use takeaway food delivery apps, those who did had 84% greater likelihood of living with obesity (RRR 1.84, 95% CI 1.20 to 2.82). There was weak evidence of a positive association between the use of takeaway food delivery apps and living with overweight (RRR 1.45, 95% CI 0.95 to 2.20). Results were similar in models adjusting for social grade (table 4) and household income (online supplemental material table S3).

**Table 4:** Adjusted associations between online delivery service use and weight status

	Online grocery delivery				Online takeaway delivery			
	Overweight		Obesity		Overweight		Obesity	
	RRR (95% CI)	P	RRR (95% CI)	P	RRR (95% CI)	P	RRR (95% CI)	P
Delivery service use	0.82 (0.55 to 1.22)	0.32	1.33 (0.91 to 1.97)	0.15	1.45 (0.95 to 2.20)	0.08	1.84 (1.20 to 2.82)	0.01
Social grade*								
High (AB)	Ref		Ref		Ref		Ref	
Middle-high (C1)	1.40 (0.99 to 1.97)	0.06	1.81 (1.23 to 2.68)	<0.01	1.40 (0.99 to 1.97)	0.06	1.77 (1.20 to 2.62)	<0.01
Middle-low (C2)	1.34 (0.86 to 2.08)	0.20	2.23 (1.39 to 3.59)	<0.01	1.32 (0.85 to 2.06)	0.22	2.17 (1.35 to 3.49)	<0.01
Low (DE)	1.21 (0.78 to 1.87)	0.40	1.72 (1.07 to 2.78)	0.03	1.18 (0.76 to 1.82)	0.46	1.61 (0.99 to 1.84)	0.05
Sex								
Female	Ref		Ref		Ref		Ref	
Male	1.50 (1.10 to 2.04)	0.01	1.35 (0.97 to 1.88)	0.08	1.52 (1.12 to 2.07)	0.01	1.33 (0.95 to 1.84)	0.09
Age (years)								
20–29	Ref		Ref		Ref		Ref	
30–39	1.25 (0.60 to 2.59)	0.56	1.24 (0.54 to 2.85)	0.61	1.28 (0.61 to 2.66)	0.52	1.32 (0.57 to 3.04)	0.52
40–49	1.44 (0.71 to 2.95)	0.31	2.22 (1.01 to 4.90)	0.05	1.50 (0.73 to 3.07)	0.27	2.48 (1.12 to 5.52)	0.03
50–59	2.80 (1.38 to 5.72)	0.01	3.03 (1.37 to 6.72)	0.01	3.05 (1.48 to 6.27)	<0.01	3.51 (1.56 to 7.88)	<0.01
60–69	4.26 (1.94 to 9.35)	<0.001	3.74 (1.55 to 9.03)	<0.01	4.68 (2.11 to 10.37)	<0.001	4.43 (1.81 to 10.85)	<0.01
70+	5.53 (2.23 to 13.68)	<0.001	3.81 (1.38 to 10.49)	0.01	6.04 (2.42 to 15.09)	<0.001	4.51 (1.61 to 12.59)	<0.01
Number of adults	1.07 (0.90 to 1.26)	0.45	1.06 (0.89 to 1.26)	0.54	1.06 (0.89 to 1.25)	0.52	1.05 (0.88 to 1.25)	0.58
Number of children	1.22 (1.00 to 1.49)	0.05	1.07 (0.87 to 1.33)	0.52	1.21 (0.99 to 1.48)	0.06	1.07 (0.86 to 1.32)	0.62
Employment								
Full time	Ref		Ref		Ref		Ref	
Part time	0.91 (0.59 to 1.40)	0.68	0.58 (0.35 to 0.95)	0.03	0.94 (0.61 to 1.45)	0.79	0.60 (0.36 to 0.99)	0.05
Self-employed	0.79 (0.47 to 1.33)	0.37	0.85 (0.50 to 1.46)	0.56	0.80 (0.48 to 1.35)	0.41	0.88 (0.51 to 1.50)	0.63
Retired	0.69 (0.39 to 1.22)	0.20	0.81 (0.44 to 1.50)	0.51	0.70 (0.40 to 1.24)	0.22	0.83 (0.45 to 1.54)	0.55
Looking after home/family	0.85 (0.45 to 1.59)	0.60	1.31 (0.71 to 2.40)	0.39	0.88 (0.47 to 1.66)	0.69	1.40 (0.76 to 2.57)	0.28
Other†	1.65 (0.95 to 2.86)	0.08	1.62 (0.92 to 2.87)	0.09	1.64 (0.95 to 2.84)	0.08	1.72 (0.97, 3.04)	0.06
Region								
North	Ref		Ref		Ref		Ref	
London	0.71 (0.54 to 0.93)	0.01	0.72 (0.54 to 0.97)	0.03	0.70 (0.53 to 0.91)	0.01	0.73 (0.54 to 0.97)	0.03
Number of observations	1245				1245			
Log likelihood	−1298.43				−1297.03			

Estimates were obtained from multinomial logistic regression models with having underweight and healthy weight (BMI <25 kg/m2) as reference category. Digital grocery refers to use of online delivery services in the previous month, digital takeaway to the use of online takeaway delivery in the past 7 days days. Living with uUnderweight and healthy weight is defined as BMI <25 kg/m2, overweight as kg/m2overweight as BMI 25–29.9 kg/m2, and obesity as BMI ≥30 kg/m2.

* Social grade was based on the National Readership Survey classification (National Readership Survey, 2018): High (AB: higher and intermediate managerial, administrative and professional), middle-high (C1: supervisory, clerical and junior managerial, administrative and professional), middle-low (C2: skilled manual workers), and low (DE: semi-skilled and unskilled manual workers, state pensioners, casual and lowest grade workers, unemployed with state benefits only);

† On a government -sponsored training scheme; working paid or unpaid for your own or family’s business; away from work ill, on maternity leave, on holiday or temporarily; laid off; doing any other kind of paid work; retired; student; long term sick or disabled; actively looking for paid work; unemployed and not looking for work; none of the above.

RRRrelative risk ratio

## Discussion

In this study, we investigated associations between social position and the use of online food delivery services for both groceries and takeaway food, and whether using these services was associated with weight status. The results of our analyses indicate that not all groups of consumers use these services equally. Purchasing groceries online was more likely among households with higher income while ordering takeaway food online was more likely among households with lower social grade. We also found that takeaway food delivery usage was associated with greater likelihood of living with obesity. We observed no association between social grade and online grocery delivery service use, income and takeaway food delivery app use, and between online grocery purchasing and weight status.

### Comparison with other studies

There are a limited number of other studies in the field. We observed a similar proportion of participants reporting takeaway food delivery apps use (13.2%) as the UK sample in one other study (15.9%).^15^ The associations between indicators of social position and online grocery delivery service use observed in the present study are in line with previous research.^31^ In contrast to the association between social grade and takeaway food delivery app use observed in this study, a previous study found a less clear pattern in the UK.^15^ This may be due to different indicators of social position used, as the former study examined education instead of social grade.^15^ We observed an association between takeaway food delivery app use and weight status, which tallies with findings from Australia,^16^ but is contrary to research conducted in the UK which did not find a relationship.^15 32^ The difference in findings may be explained by the different geographical locations (London and the north of England vs Scotland and England) and sample characteristics (eg, compared with our sample, the Scottish sample was younger, and BMI was below population average in the English study). Though not the focus of this study, we found that the use of online grocery delivery services was associated with age and gender, and the use of takeaway food delivery apps with age, which is in line with previous research.^15 31^

### Interpretation

Our findings suggest that there are differences in use, both within and between the online grocery and online takeaway food sector. The use of online grocery delivery services was higher among the most affluent households, while takeaway food delivery app use was higher among households with lower social position. Dietary quality of food purchases was not measured in this study, but previous research indicates that takeaway food delivery app use is associated with lower dietary quality and that foods purchased from takeaways are more energy dense and nutrient poor.^17^ While there is mixed evidence on the healthfulness of online grocery shopping, as consumers both tend to be more hesitant in buying perishable foods and are less prone to impulse purchases and tend to buy fewer discretionary foods,^11^ studies indicate that overall, online shopping baskets tend to be of higher dietary quality compared with in-store purchasing.^9 10^ The differential use of these services by social position observed in the present study may lead to a widening of dietary inequalities. Future research is needed to ascertain implications on diet and dietary health arising from the differential usage of online food delivery services observed in this study.

In the grocery sector, it has been hypothesised that a shift to online grocery shopping will occur more rapidly among affluent households^33^ and it is possible that we observed evidence for this. More affluent households have the financial capacity to meet minimum spend requirements for grocery delivery, pay delivery costs, and take advantage of the cost savings associated with bulk purchasing through greater storage space in homes. Previous research has indicated that purchasing groceries online is associated with healthier food choices.^9 10^ In turn, differences in the use of online grocery delivery services may widen dietary inequalities by further benefitting households with higher incomes compared to those with lower incomes.^4 34^

Within the takeaway food sector, increasing availability of takeaway food delivery services has expanded the number of restaurants and fast-food outlets able to offer delivery, increased the reach of individual restaurants and meal options available to consumers, and reduced the effort required to access takeaway food.^14^ In contrast to online grocery purchasing, takeaway food delivery app use was not associated with income, but with lower social grade instead. These differential observations suggest that the chosen two indicators capture different dimensions of social position which have different meanings for the use of online food delivery services. Potentially, grocery purchasing may predominantly depend on financial resources, while takeaway purchasing may be linked to culture and social group.^35^

Our findings are corroborated by a previous UK study which found that consumers with lower socioeconomic status purchased fast food more frequently in comparison to consumers with high socioeconomic status, while the latter purchased meals from restaurants more frequently and had a greater overall spend.^29^ In addition, more deprived areas, as defined through the English Indices of Multiple Deprivation, had greater online access to takeaway food, operationalised as the number of food outlets accessible through online channels in a given area, in England^36^; this suggests that access to predominantly unhealthy food may be amplified through digital channels in more deprived areas which already have greater exposure to an unhealthy food environment.^37^ However, this pattern was observed to be reversed in London^38^ and urban centres elsewhere.^8^

Consumption of fast food and takeaway food has previously been associated with excess energy intake and higher body weight.^20 39 40^ Recent studies showed that food provided by major UK restaurant chains failed to meet public health recommendations.^41^,^43^ Further research is needed to identify the mechanisms behind the observed positive association between the use of takeaway food delivery apps and weight status.

### Limitations

There are limitations to our study. First, this is a cross-sectional study, which prevents the establishment of causal associations. It provides only a snapshot of online purchasing, which may occur less than weekly (in the case of takeaway food) or monthly (in the case of groceries) as investigated in this study. While take-home purchase data were available over time, we used only 1 month to ensure time comparability with the survey data. Survey responses may also be subject to recall bias and social desirability bias, whereby individuals either forgot occasions or intentionally reported fewer occasions of takeaway food delivery app use, resulting in underestimated service use. While purchase data are more objective compared with dietary recalls,^44^ households may have failed to report all purchases. Our analyses were also limited by the uneven distribution of households across social grade and income groups. Finally, our sample of predominantly urban households in London and the north of England may not be fully representative of England.

## Conclusions

Usage of online food delivery services was patterned by social position, with differing associations observed according to the marker of social position used. Purchasing groceries online was more likely among households with higher income, while purchasing takeaway food online was more likely among those with lower occupational social grade. Takeaway food delivery app use was positively associated with living with obesity. The potential impact of increased and differential usage of online delivery services on diet and dietary inequalities warrants further research.

## supplementary material

10.1136/bmjph-2023-000487 online supplemental file 1

## Data Availability

Data may be obtained from a third party and are not publicly available.
