# Supplementary material for: Social inequalities in the use of online food delivery services and associations with weight status: cross-sectional analysis of survey and consumer data
Source: BMJ Public Health. 2024 Jul 24;2(2):e000487. doi: 10.1136/bmjph-2023-000487 (PMC11816591; doi:10.1136/bmjph-2023-000487)
Supplement: online supplemental file 1 [file bmjph-2-2-s001.pdf]

Table S1 Associations between missing data for income and online food delivery service use

	Online grocery delivery service use						Online takeaway delivery app use					
	Unadjusted			Adjusted			Unadjusted			Adjusted		
	**OR**	**p-value**	**95%CI**	**OR**	**p-value**	**95%CI**	**OR**	**p-value**	**95%CI**	**OR**	**p-value**	**95%CI**
**Food delivery service use**	0.99	0.95	0.67, 1.45	0.99	0.96	0.66, 1.48	0.61	0.04	0.38, 0.98	0.76	0.28	0.46, 1.25
**Social grade**												
High (AB)				*Ref*						*Ref*		
Middle-high (C1)				0.96	0.82	0.66, 1.38				0.96	0.82	0.66, 1.39
Middle-low (C2)				0.86	0.52	0.53, 1.38				0.86	0.55	0.54, 1.39
Low (DE)				0.93	0.76	0.59, 1.47				0.95	0.83	0.60, 1.50
**Sex**												
Female				*Ref*						*Ref*		
Male				0.62	0.01	0.44, 0.88				0.63	0.01	0.44, 0.89
**Age**												
20–29 years				*Ref*						*Ref*		
30–39 years				2.32	0.19	0.67, 8.08				2.27	0.20	0.65, 7.93
40–49 years				3.15	0.06	0.94, 10.54				3.06	0.07	0.91, 10.25
50–59 years				3.15	0.06	0.94, 10.50				2.99	0.08	0.89, 10.02
60–69 years				3.81	0.04	1.09, 13.28				3.58	0.05	1.02, 12.56
70+ years				2.78	0.13	0.74, 10.49				2.62	0.16	0.69, 9.94
**Number of Adults**				1.21	0.02	1.03, 1.43				1.22	0.02	1.03, 1.43
**Number of Children**				0.87	0.23	0.69, 1.09				0.87	0.24	0.69, 1.10

**Employment**												
Full time				*Ref*						*Ref*		
Part time				0.80	0.39	0.47, 1.34				0.79	0.36	0.47, 1.32
Self-employed				1.28	0.37	0.75, 2.19				1.27	0.38	0.74, 2.17
Retired				1.89	0.03	0.75, 2.19				1.87	0.03	1.07, 3.27
Looking after home/family				1.67	0.08	0.94, 2.96				1.64	0.09	0.92, 2.91
Other				1.01	0.97	0.55, 1.86				1.00	0.99	0.55, 1.84
**Region**												
North				*Ref*						*Ref*		
London				1.34	0.05	1.01, 1.79				1.35	0.04	1.01, 1.80
Number of observations	1,521			1,521			1,521			1,521		
Log likelihood	-651.29			-624.54			-648.99			--623.92		

95%CI = 95% confidence interval; OR = Odds Ratio. Estimates from logistic regressions modelling the odds of missing observations for household income. Online grocery delivery refers to use of online delivery services in the previous month, online takeaway delivery to the use of online takeaway delivery apps in the past 7 days.

Table S2 Associations between missing data for weight status and online food delivery service use

	Online grocery delivery service use						Online takeaway delivery app use					
	Unadjusted			Adjusted			Unadjusted			Adjusted		
	OR	p-value	95%CI	OR	p-value	95%CI	OR	p-value	95%CI	OR	p-value	95%CI
**Food delivery service use**	1.29	0.14	0.92, 1.82	1.18	0.35	0.83, 1.68	0.87	0.50	0.58, 1.30	0.76	0.20	0.50, 1.15
**Social grade**												
High (AB)				*Ref*						*Ref*		
Middle-high (C1)				1.15	0.42	0.81, 1.64				1.15	0.44	0.81, 1.63
Middle-low (C2)				0.85	0.48	0.54, 1.34				1.52	0.52	0.55, 1.36
Low (DE)				1.11	0.65	0.71, 1.72				1.13	0.60	0.72, 1.75
**Sex**												
Female				*Ref*						*Ref*		
Male				0.68	0.02	0.49, 0.94				0.67	0.02	0.48, 0.93
**Age**												
20–29 years				*Ref*						*Ref*		
30–39 years				1.31	0.48	0.62, 2.75				1.28	0.51	0.61, 2.70
40–49 years				1.55	0.23	0.76, 3.14				1.52	0.25	0.75, 3.09
50–59 years				1.28	0.49	0.63, 2.61				1.22	0.59	0.59, 2.49
60–69 years				0.99	0.98	0.45, 2.21				0.93	0.85	0.41, 2.07
70+ years				0.68	0.46	0.25, 1.86				0.64	0.38	0.23, 1.75
**Number of Adults**				1.36	<0.001	1.17, 1.58				1.36	<0.001	1.17, 1.58
**Number of Children**				0.95	0.56	0.79, 1.14				0.95	0.61	0.79, 1.45

**Employment**												
Full time				*Ref*						*Ref*		
Part time				0.82	0.36	0.54, 1.25				0.81	0.32	0.53, 1.23
Self-employed				0.75	0.28	0.45, 1.26				0.75	0.27	0.45, 1.25
Retired				0.78	0.42	0.43, 1.43				0.78	0.42	0.43, 1.43
Looking after home/family				1.20	0.49	0.72, 2.00				1.16	0.56	0.70, 1.95
Other				0.90	0.68	0.54, 1.50				0.91	0.73	0.55, 1.52
**Region**												
North				*Ref*						*Ref*		
London				0.94	0.65	0.72, 1.23				0.95	0.70	0.72, 1.24
Number of observations	1,521			1,521			1,521			1,521		
Log likelihood	-719.31			-693.47			-720.11			-693.03		

95%CI = 95% confidence interval; OR = Odds Ratio. Estimates from logistic regressions modelling the odds of missing observations for main reporter's weight status. Online grocery delivery refers to use of online delivery services in the previous month, online takeaway delivery to the use of online takeaway delivery apps in the past 7 days.

Table S3 Adjusted associations between online food delivery service use and overweight and obesity, adjusted for household income

	Online grocery delivery service use						Online takeaway delivery app use					
**Weight status**	**Overweight**			**Obesity**			**Overweight**			**Obesity**		
	**RRR**	**p-value**	**95%CI**	**RRR**	**p-value**	**95%CI**	**RRR**	**p-value**	**95%CI**	**RRR**	**p-value**	**95%CI**
**Food delivery service use**	0.84	0.41	0.55, 1.28	1.29	0.22	0.86, 1.95	1.29	0.26	0.83, 2.00	1.80	0.01	1.16, 2.80
**Income**												
Up to £19,999 p.a.	*Ref*			*Ref*			*Ref*			*Ref*		
£20,000–49,999 p.a.	1.04	0.19	0.71, 1.50	1.09	0.65	0.74, 1.62	1.03	0.88	0.71, 1.49	1.10	0.62	0.75, 1.63
£50,000 or more p.a.	1.19	0.46	0.74, 1.92	0.80	0.39	0.48, 1.34	1.18	0.70	0.74, 1.90	0.84	0.50	0.50, 1.40
**Sex**												
Female	*Ref*			*Ref*			*Ref*			*Ref*		
Male	1.46	0.02	1.06, 2.03	1.28	0.17	0.90, 1.81	1.49	0.02	1.07, 2.06	1.26	0.20	0.89, 1.78
**Age**												
20–29 years	*Ref*			*Ref*			*Ref*			*Ref*		
30–39 years	1.09	0.82	0.52, 2.30	1.18	0.70	0.51, 2.73	1.11	0.79	0.52, 2.34	1.25	0.60	0.54, 2.90
40–49 years	1.28	0.51	0.62, 2.64	1.94	0.10	0.87, 4.31	1.30	0.48	0.63, 2.70	2.13	0.07	0.95, 4.76
50–59 years	2.74	0.01	1.33, 5.64	2.94	0.01	1.32, 6.56	2.88	0.01	1.39, 5.98	3.35	<0.01	1.49, 7.57
60–69 years	4.15	<0.01	1.85, 9.34	3.27	0.01	1.33, 8.06	4.41	<0.001	1.95, 10.01	3.84	<0.01	1.54, 9.57
70+ years	5.46	<0.01	2.10, 14.23	2.67	0.07	0.93, 7.69	5.74	<0.001	2.19, 15.06	3.12	0.04	1.07, 9.10
**Number of Adults**	1.03	0.72	0.86, 1.24	1.07	0.47	0.89, 1.29	1.03	0.98	0.63, 1.60	1.06	0.54	0.88, 1.28
**Number of Children**	1.25	0.04	1.01, 1.54	1.05	0.67	0.84, 1.31	1.24	0.05	1.01, 1.53	1.04	0.74	0.83, 1.30
**Employment**												
Full time	*Ref*			*Ref*			*Ref*			*Ref*		
Part time	0.99	0.95	0.62, 1.57	0.69	0.17	0.41, 1.17	1.01	0.98	0.63, 1.60	0.71	0.21	0.42, 1.21
Self-employed	0.76	0.35	0.43, 1.34	0.94	0.83	0.53, 1.65	0.78	0.38	0.44, 1.37	0.97	0.92	0.55, 1.71
Retired	0.71	0.29	0.38, 1.34	1.01	0.97	0.52, 1.99	0.72	0.31	0.38, 1.36	1.03	0.93	0.52, 2.02
Looking after home/family	0.92	0.82	0.46, 1.85	1.48	0.24	0.77, 2.85	0.94	0.86	0.47, 1.88	1.60	0.17	0.83, 3.08

Other[1]	1.57	0.12	0.89, 2.80	1.70	0.07	0.95, 3.05	1.55	0.13	0.87, 2.74	1.77	0.05	0.99, 3.18
**Region**												
North	*Ref*			*Ref*			*Ref*			*Ref*		
London	0.72	0.03	0.54, 0.96	0.78	0.12	0.57, 1.06	0.71	0.02	0.53, 0.96	0.78	0.12	0.57, 1.06
Number of observations	1,101						1,101					
Log likelihood	-1,154.24						-1,152.66					

95%CI = 95% confidence interval; RRR = Relative Risk Ratio. Estimates were obtained from multinomial logistic regression models with having underweight and healthy weight (BMI < 25 kg/m^2) as reference category. Digital grocery refers to use of online delivery services in the previous month, digital takeaway to the use of online takeaway delivery in the past 7 days. Living with underweight and healthy weight is defined as BMI < 25 kg/m^2, overweight as 25 kg/m^2 ≤ BMI > 30 kg/m^2, and obesity as BMI ≥ 30 kg/m^2. [1]On a government sponsored training scheme; working paid or unpaid for your own or family's business; away from work ill, on maternity leave, on holiday or temporarily; laid off; doing any other kind of paid work; retired; student; long term sick or disabled; actively looking for paid work; unemployed and not looking for work; none of the above
